# Work-Related Factors and Lung Cancer Survival: A Population-Based Study in Switzerland (1990–2014)

**DOI:** 10.3390/ijerph192113856

**Published:** 2022-10-25

**Authors:** Nicolas Bovio, Michel Grzebyk, Patrick Arveux, Jean-Luc Bulliard, Arnaud Chiolero, Evelyne Fournier, Simon Germann, Isabelle Konzelmann, Manuela Maspoli, Elisabetta Rapiti, Irina Guseva Canu

**Affiliations:** 1 Center for Primary Care and Public Health (Unisanté), University of Lausanne, 1010 Lausanne, Switzerland; 2 Department of Occupational Epidemiology, National Research and Safety Institute (INRS), 54500 Vandoeuvre lès Nancy, France; 3 Neuchâtel and Jura Cancer Registry, 2000 Neuchâtel, Switzerland; 4 Population Health Laboratory, University of Fribourg, 1700 Fribourg, Switzerland; 5 Valais Cancer Registry, Valais Health Observatory, 1950 Sion, Switzerland; 6 Institute of Primary Health Care (BIHAM), University of Bern, 3012 Bern, Switzerland; 7 School of Population and Global Health, McGill University, Montréal, QC H3A 1G1, Canada; 8 Geneva Cancer Registry, University of Geneva, 1211 Geneva, Switzerland

**Keywords:** net survival, lung cancer, occupation, Switzerland, gender differences, workers

## Abstract

While previous Swiss studies have demonstrated differences in lung cancer mortality between occupational groups, no estimates are available on the association of occupation-related factors with lung cancer survival. This study aimed at determining whether occupation or work-related factors after diagnosis affect lung cancer survival. We used cancer registry records to identify lung cancer patients diagnosed between 1990 and 2014 in western Switzerland (*n* = 5773) matched with the Swiss National Cohort. The effect of occupation, the skill level required for the occupation, and the socio-professional category on 5-year lung cancer survival was assessed using non-parametric and parametric methods, controlling for histological type and tumour stage. We found that the net survival varied across skill levels and that the lowest skill level was associated with worse survival in both men and women. In the parametric models with minimal adjustment, we identified several occupational groups at higher risk of mortality compared to the reference category, particularly among men. After adjustment for histological type of lung cancer and tumour stage at diagnosis, most hazard ratios remained higher than 1, though non-statistically significant. Compared to top managers and self-employed workers, workers in paid employment without specific information on occupation were identified as the most at-risk socio-professional category in nearly all models. As this study was conducted using a relatively small sample and limited set of covariates, further studies are required, taking into account smoking habits and administrated cancer treatments. Information on return to work and working conditions before and after lung cancer diagnosis will also be highly valuable for analysing their effect on net lung cancer survival in large nationwide or international studies. Such studies are essential for informing health and social protection systems, which should guarantee appropriate work conditions for cancer survivors, beneficial for their quality of life and survival.

## 1. Introduction

Work is an important health determinant and occupation is a key variable in occupational health. Occupation enables estimating or approximating the working conditions and occupational exposures to carcinogenic substances and analysing their effect on incidence and mortality from specific causes of death, including cancer. Occupation influences both incidence and mortality for several types of cancer. In Switzerland, we recently demonstrated its effect on the incidence of breast cancer in working women and on the stage of breast cancer at diagnosis [1], and on the mortality from lung cancer in the Swiss working population [2,3]. Lung cancer has a poor prognosis and results in the highest mortality among all cancers, with 1.8 million deaths worldwide in 2020 [4,5]. Therefore, the differences in incidence and mortality rates across different occupational groups are usually similar for lung cancer.

While the effect of occupation and occupational exposure to lung carcinogens has been well demonstrated for lung cancer incidence and mortality [6,7,8,9], little is known about the effect of work-related factors on lung cancer survival. The five-year survival of lung cancer varies between 10% and 20% worldwide [10]. However, thanks to important advances in the treatment and diagnosis of lung cancer in the past 10 years, lung cancer survival has seen the first improvements [11]. In view of these improvements, the number of cancer survivors is increasing and the ability to return to work after cancer is now considered a realistic goal [12].

Work plays an important role in lung cancer survival [13]. A better survival rate was found in stage III and IV lung cancer patients who had returned to work [14]. However, the return to work among cancer survivors is not always possible due to sociodemographic, work-related, and clinical-related factors. Lung cancer survivors suffer from severe fatigue and have a worse health-related quality of life compared with other cancer survivors [15]. Moreover, lung cancer survivors experience a high risk of unemployment and very low professional reintegration after an interruption due to illness [16,17,18]. In Italy, a significant association between job loss and gender (male), low level of education, heavy work, advanced stage of cancer, chemotherapy, and comorbidity was reported [17]. This is also true in Japan, although there, female gender constitutes a disadvantage in the return to work [19]. In South Korea, compared to patients returning to work, unemployed cancer survivors are older, likely manual/service-oriented workers, have lower family income, have undergone chemotherapy, have fewer unmet health system and information needs, poorer physical functioning, and negative illness perceptions [20,21]. Cancer survivors able to return to work usually have a less stable employment trajectory than other workers [18] and may face financial problems due to a reduction in working hours [12]. Although the financial distress characteristic of cancer patients seems to be primarily determined by pre-diagnosis sociodemographic factors [22], financial and occupation-related factors that might change during the course of the disease can negatively affect the quality of life and survival [23,24].

A recent systematic review and meta-analysis explored the effect on survival of different socioeconomic measures including education, income, and area-based socio-economic status most often used as an index [25]. However, the effect of occupation could not be explored in the meta-analysis because of the heterogeneity of the measures across studies [25]. Eight studies included in the narrative synthesis used individual data, four designated occupational status by collar colour or similar categories [26,27,28,29], one was based on socio-professional categories [30], and three studies provided more details on occupation, but only specific groups were analysed [31,32,33]. Most of the included studies being one or several decades old, there is a need not only to analyse survival using standardized occupation variables but also to update lung cancer survival estimates for work-related factors.

In Switzerland, the relative survival of lung cancer and its determinants were assessed by Galli et al. [34]. The authors reported that the five-year survival was 24% for women and 19% for men, while the ten-year survival was 15% and 11%, respectively [34]. The higher the stage at diagnosis, and the older the patient, the lower the survival, in both men and women. Patients with small cell lung cancer also had lower survival compared to those with non-small cell histological sub-type. However, the relationship between occupation or work-related variables and lung cancer survival was not assessed [34].

### Study Objectives

Since previous Swiss studies have demonstrated differences in lung cancer mortality between occupational groups, we aimed at determining whether occupation or work-related factors after diagnosis affect lung cancer survival. The objective of this study was therefore to investigate this association by focusing on (1) occupation, (2) skill level required for the occupation, and (3) socio-professional categories independently, using relative survival settings. 

## 2. Materials and Methods

### 2.1. Sources of Data

We used data from the cancer registries of western Switzerland (cantons of Geneva, Neuchâtel, Vaud, and Valais) for the period 1990–2014. In order to retrieve information on occupation and mortality, we used data from the Swiss National Cohort (SNC), with an estimated population coverage of 98.6% [35]. The SNC is based on data from the 1990 and 2000 federal censuses, which were linked to mortality, birth, and emigration records. In order to have a single database, the registries transmitted all their lung cancer cases diagnosed in the study period to the Centre for Primary Care and Public Health (Unisanté). Unisanté centralized the registries’ data and harmonized their format to enable linkage with SNC data. The linkage between the cancer registries and SNC was carried out by the Institute of Social and Preventive Medicine of the University of Bern and almost all patients in the cancer registries could be linked with the SNC data (94.4%).

### 2.2. Study Sample and Follow-Up

In Switzerland, the minimum legal working age is 15 and the age of majority is 18. The legal retirement age is 65 for men and 64 for women. Therefore, our study sample included lung cancer cases aged between 18 and 65 years at the time of either the 1990 or 2000 census, with a known occupation. Patients were followed from the date of lung cancer diagnosis until the earliest of the following events: date of emigration, 85th birthday, death, or study termination (31 December 2014).

### 2.3. Definition of Predictor Variables

We selected three different work-related variables to assess their association with lung cancer survival. The first is the participant’s occupation, which was collected twice (in 1990 and 2000) and coded according to the International Standard Classification of Occupations, 1988 version (ISCO-88) established by the International Labour Organization (ILO). This multi-tiered classification was used in both censuses and initially coded in four-digit codes by the Swiss Federal Statistical Office (SFSO). For this study, we used the first digit of the ISCO-88 code, which identifies nine major occupational groups (e.g., legislators, senior officials and managers, clerks, technicians, and associated professionals). The second variable is the skill level required for the occupation, also established by the ILO based on ISCO, version 2008 (ISCO-08) [36,37]. To create this variable, we recoded occupations according to the ISCO-08, using an official crosswalk between ISCO-88 and ISCO-08 operated by the PROCODE software [38]. The variable was then created as follows: Occupations grouped within the ISCO-08 major code 9—Elementary occupations were classified as Skill level 1 (i.e., occupations that require simple and routine physical or manual tasks). Skill level 2 (i.e., occupations that involve the performance of tasks such as operating machinery and electronic equipment; driving vehicles; maintenance and repair of electrical and mechanical equipment; and manipulation, ordering and storage of information) encompassed occupations grouped within the ISCO-08 major codes 4—Clerical support workers (six occupations), 5—Service and sales workers (nine occupations), 6—Skilled agricultural, forestry, and fishery (six occupations), 7—Craft and related trades workers (twenty occupations), and 8—Plant and machine operators, and assemblers (twelve occupations). Skill level 3 (i.e., occupations that involve the performance of complex technical and practical tasks that require an extensive body of factual, technical, and procedural knowledge in a specialised field corresponded to the ISCO major group 3—Technicians and associate professionals, including 10 occupations. Finally, 5 occupations grouped within the ISCO-08 major code 1—Managers, and 15 occupations in the ISCO-08 code 2—Professionals corresponded to the occupations with Skill level 4 (i.e., occupations that typically involve the performance of tasks which require complex problem-solving and decision making based on an extensive body of theoretical and factual knowledge in a specialised field). The third measure is the socio-professional category, a composite of occupation, occupational status, the highest level of education completed, and legal form of business defined according to the SFSO [39]. This is a six-class variable helping distinguish workers having top management and independent professions; other self-employed workers; professionals and senior management workers; supervisors with low-level management positions and skilled labourers; unskilled employees; and workers in paid employment, not classified elsewhere.

As start and end dates of employment were not known, we assigned the 1990 census occupational information for patients diagnosed with lung cancer between 1990 and 2000, and the 2000 information thereafter.

### 2.4. Case Selection, Tumour Stage and Histological Type

We considered primary malignant lung cancer (C33-C34) based on the International Classification of Diseases for Oncology (ICD-O-3), 3rd edition. We identified cases using the four western Swiss cancer registries and applied the International Agency for Research on Cancer (IARC) rules for multiple primary cancers [40]. The histological types were grouped into the following categories: squamous cell carcinoma (8052, 8070–8076, 8083, 8123), small cell carcinoma (8002, 8041–8045), adenocarcinoma (8050, 8140, 8144, 8250, 8253, 8255, 8260, 8290, 8310, 8323, 8480, 8481, 8490, 8550), large cell carcinoma (8012, 8013, 8021, 8082), other specified carcinoma (8003, 8004, 8022, 8031, 8033, 8200, 8240, 8241, 8244, 8246, 8249, 8430, 8560), unspecified malignant neoplasms (8000, 8001, 8010, 8020, 8030, 8046) and unclassifiable [41].

The tumour stage at diagnosis was coded by cancer registries according to the classification of malignant tumours (TNM) [42]. Tumours localized to the organ of origin constituted stages I and II, locally extensive spread, particularly to regional lymph nodes, stage III, and tumours with distant metastasis, stage IV. The stage at diagnosis was imputed, when missing, with multivariate imputation by chained equations [43] using the following variables as predictors: age at diagnosis, survival time, histological type of lung cancer, status at the end of follow-up (censored or dead), the skill level required for the occupation, socio-professional category, cancer registry, language region, and civil status. We ran all models with 25 imputations, in order to reduce the impact of the random sampling inherent in multiple imputation procedures [44]. Comparison of the proportions of each category of the stage between the observed and imputed data showed a better match by grouping stages III and IV in one category. Consequently, we grouped stages III and IV and considered the stage as a three-class variable.

### 2.5. Statistical Analyses

Net survival can be used to estimate the survival that would be observed if the only possible underlying cause of death was the disease under study [45]. It can be calculated using either the cause-specific or relative survival approach. Prior findings showed that the latter was more robust and recommended for net survival analysis [46]. In this setting, net survival is estimated using life tables and can be defined as the ratio of the observed survival to the one expected from the life tables. In other words, it approximates the net survival probability and can be seen as the survival probability from the disease under study after all other risks have been removed [47]. 

In this study, we applied two methods based on relative survival settings. First, the Pohar–Perme nonparametric method [48] with the log-rank type test was applied to compare the net survival curves between groups [49]. Secondly, we applied a parametric method that models the excess hazard in a framework of multivariable proportional hazard regression model [50]. Both analyses were conducted separately for men and women, as recommended by the European Agency for Safety and Health at Work [51].

### 2.6. Nonparametric Survival Analysis

For the nonparametric approach, we used the STNS package developed in STATA [52]. It requires the all causes of mortality rate table, which is used to compute the expected hazard and survival of each subject at each event time in the dataset. We calculated it using the mortality rates of the population of the cantons of Geneva, Neuchâtel, Vaud, and Valais stratified by 5-year age group (18–85 years) and 5-year calendar period (1990–2014). These categories were chosen to smooth the rates and avoid large differences in mortality by age or calendar year. Since we were mainly interested in the survival by occupation, we also stratified our rates by occupation. This allowed us to account for differences in overall mortality between occupations aggregated at 1-digit of the ISCO-88. Net lung cancer survival was then computed at 5 years by occupation, skill level, and socio-professional category, independently. We applied a log-rank test to compare the net survival curves between groups.

### 2.7. Multivariate Parametric Survival Analysis

For the parametric approach, we used the flexrsurv R package [53]. A cubic spline with three knots (1, 5, and 10 years of follow-up), the internal breakpoints that define the spline used to estimate the baseline hazard, were fitted. Background mortality rates were the same as in the nonparametric survival analysis. Again, we calculated the excess hazards by occupation, skill level, and socio-professional category. For each of these variables, we fitted a model adjusted for age, calendar period at diagnosis, and canton (Model 1). Then, we completed Model 1 by adding the histological type (Model 2). Finally, we added in Model 2 the tumour stage at diagnosis (Model 3). We tested the non-proportional effect of the stage using B-Splines [54]. In order to compare the fit of our models, we used the Akaike information criterion (AIC) [55]. To assess the association between tumour stage and our predictors, we also performed a Chi2 test.

## 3. Results

### 3.1. Cohort Description

Of the 13,427 lung cancer cases diagnosed between 1990 and 2014, we excluded 48% of men and 67% of women because of a lack of information on their occupations. Unemployed and job-seeking people, who represented 3% of the total, were also excluded. The final sample consisted of 5773 patients, 76% of which were men (Table 1). Most patients were Swiss, with only 22% and 16% being non-Swiss men and women, respectively. More than half of the study patients were married and about one-third were single. The mean age at diagnosis was 60.7 ± 8.0 years in men and 58.4 ± 8.5 years in women and the mean duration of follow-up was 2.5 ± 4.0 years and 2.8 ± 4.1 years, respectively. The most represented occupational group differed between men and women. In men, it was craft and related trades workers (24% versus 4% for women). In women, the main occupational group was clerks (26% compared to 8% for men). About half of the study patients had occupations requiring the second lowest level of skills. In addition, both male and female patients were more likely to be in the supervisors/low-level management and skilled labour socio-professional categories. Patients in top management and independent occupations accounted for only 5% of men and 2% of women.

The tumour stage at diagnosis was known for 61% of men and 71% of women (Table 2). Participants were more likely to be diagnosed at lung cancer stage IV, with 33% in men and 39% in women. After multiple imputation of the missing values, we observed no association between the tumour stage and occupation or skill level (result not shown). Conversely, the tumour stage was associated with socio-professional category but only in men (*p* = 0.03) (result not shown). In both sexes, adenocarcinoma was the most common histological type of lung cancer (33% in men and 48% in women) (Table 2). In men, squamous cell carcinoma and small cell carcinoma accounted for 29% and 16% of all cancers, and in women for 11% and 16%, respectively.

### 3.2. Five-Year Net Survival per Occupation

**Figure 1:**
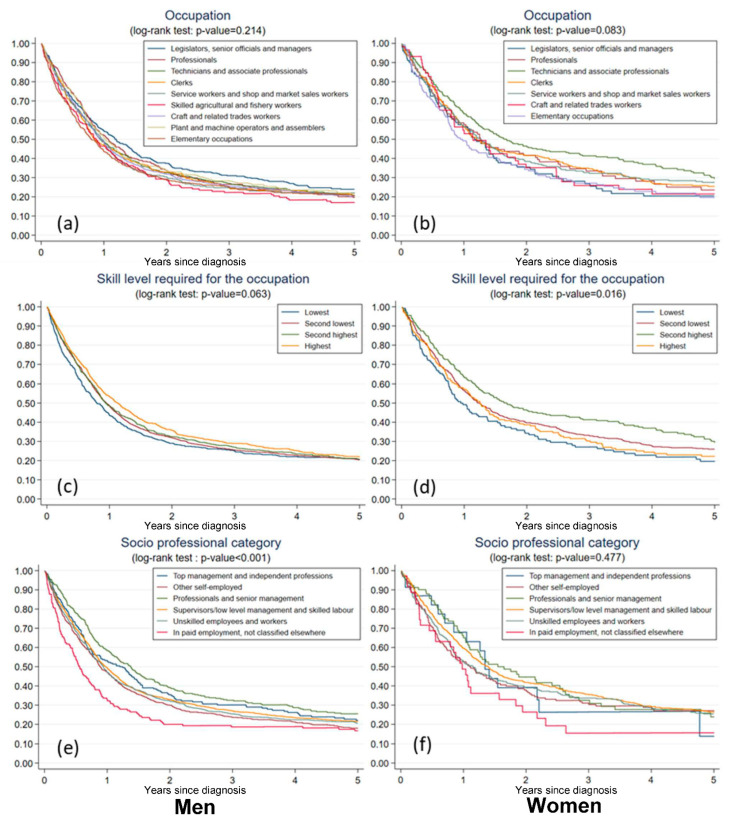
Nonparametric five-year net survival of lung cancer in men and women across (**a**,**b**) occupation, (**c**,**d**) skill level required for the occupation, and (**e**,**f**) socio-professional category.

In the nonparametric setting, the overall log-rank test showed no difference in lung cancer survival across occupational groups in men (*p* = 0.21) (Figure 1a). However, legislators, senior officials, and managers had the highest 5-year net survival (0.24, 95% CI: 0.20–0.28), whereas skilled agricultural and fishery workers had the lowest net survival (0.17, 95% CI: 0.11–0.23). In the parametric setting, several occupations exhibited an excess hazard when compared to legislators, senior officials, and managers (Table 3). Service workers and shop and market sales workers, skilled agricultural and fishery workers, and workers in elementary occupations were the three male occupational groups with the highest hazard ratios ranging between 1.17 and 1.23 (Table 3, Model 1). The additional adjustment for histological type (Model 2) and for tumour stage (Model 3) did not modify this rating. The hazard ratios remained greater than 1, but decreased to 1.14–1.19 in Model 2, and to 1.06–1.13 in Model 3, and became non-statistically significant. 

Among women, contrary to men, legislators, senior officials, and managers had the lowest net survival (0.20, 95%-IC: 0.12–0.29), while technicians and associate professionals experienced the highest five-year net survival (0.29, 95%-IC: 0.23–0.36) (Figure 1b). This finding was also observed in the parametric analysis. (Table 4).

### 3.3. Five-Year Net Survival per Skill Level Required for the Occupation

In the non-parametric analysis, the variations in net survival across skill levels were statistically significant in women but of borderline statistical significance in men (*p* = 0.06) (Figure 1c,d). In both genders, workers with the lowest skill level presented the lowest 5-year net survival (0.20, 95%-CI: 0.12–0.27 in women and 0.21, 95% CI: 0.17–0.24 in men). The highest 5-year survival was observed among male workers with the highest skill level (0.22, 95%-CI: 0.19–0.25) and in female workers with the second highest skill level (0.29, 95%-CI: 0.23–0.36).

These results of parametric analyses confirmed this finding (Table 3 and Table 4) 

### 3.4. Five-Year Net Survival per Socio-Professional Category

In the non-parametric approach, we found statistically significant differences in net survival across socio-professional categories in men but not in women (Figure 1e,f). Further, in both genders, workers in paid employment with unspecified occupations experienced the lowest net survival. Male professionals and senior managers had the highest five-year net survival (0.26, 95%-CI: 0.21–0.30), followed by top managers and independent workers (0.22, 95%-CI: 0.16–0.28) (Figure 1). The parametric analysis confirmed that male workers in paid employment not classified elsewhere have the highest excess risk of mortality, which remained greater than 30% and statistically significant even after adjustment for both the histological type and the tumour stage at diagnosis (Table 3). Among women, in parametric analyses, workers in paid employment not classified elsewhere were also found to be at risk, especially in the fully adjusted model, with a similar hazard ratio as in men though not statistically significant (1.41, 95%-CI: 0.75–2.67 versus 1.36, 95%-CI:1.05–1.76, respectively) (Table 4).

## 4. Discussion

### 4.1. Summary of the Main Results

In this study, we analysed the relationship between work-related factors and the five-year net survival for lung cancer. For this, we considered three complementary work-related variables: the official ILO standardized classification of occupations (ISCO-88), the skill level required for the occupation, and the socio-professional category, and we used a double analytical approach based on parametric and non-parametric analyses. To our knowledge, this study is the first to apply such a methodology to investigate the potential work-related determinants of lung cancer survival.

In the non-parametric analysis, we found that the net survival varied across skill levels and that the lowest skill level was associated with the worst survival in both men and women. In the parametric models with a minimal adjustment (Model 1), we identified several occupational groups at higher risk of mortality compared to the reference category, particularly among men. We observed that after adjustment for histological type of lung cancer and tumour stage at diagnosis, the hazard ratios increased in Model 1 and remained higher than 1, though non-statistically significantly. Finally, we demonstrated that Swiss workers in paid employment but with unspecified occupation have the worst net survival compared to other socio-professional categories, and this finding was further confirmed in the fully adjusted parametric analysis.

### 4.2. Results Interpretation

The effect of the socio-professional category on lung cancer survival, and the identification of workers in paid employment not classified elsewhere as an at-risk group in both genders appears to be a consistent finding. The classification of socio-professional categories was more exhaustive than in an earlier study and took into account all levels of the population’s socio-professional structure [30]. We observed that men in this socio-professional category were diagnosed at a later stage (stage III and IV) (81%, result not shown) than men in other socio-professional categories, but this was not true among women. The meta-analysis of the association between socio-economic status and lung cancer stage at diagnosis provided no evidence [56]. However, in the present study, the adjustment for the tumour stage had a different impact on hazard estimates in males and females belonging to this socio-professional category. In men, the fully adjusted hazard ratio was lower than the minimally adjusted one, whereas in women the former was much higher than the latter. In contrast, the adjustment for histological type of lung cancer resulted in lower estimates of hazard ratio in both genders but also in all occupational groups. Some histological types of lung cancer, namely small cell carcinoma and squamous cell carcinoma, are strongly associated with smoking [57,58,59]. Individual data on smoking were not available, hence we used the histological type of lung cancer to indirectly control for the effect of smoking, and it appeared to be a relevant strategy. Previous findings have demonstrated that smoking at the time of diagnosis was an independent predictor of reduced lung cancer survival and that the effect of smoking status was not explained by sociodemographic factors, stage, or treatment [60]. Conversely, smoking cessation at or around the time of diagnosis had a beneficial effect on the survival of lung cancer patients [61], particularly with early-stage lung cancer [62].

It is challenging to explain why workers in paid employment but with an unclassified occupation have an excess risk of mortality. One might speculate that they work in elementary occupations, which were identified as at-risk among men. However, in women, working in elementary occupations was beneficial for five-year survival. In Switzerland, elementary occupations have the highest proportion (>60%) of part-time workers, three-fourths of whom are women [63]. Depending on whether part-time work was voluntary or involuntary (when it was impossible to return to full-time work) [18], it could be either protective or a risk factor for survival. However, the evidence is very scarce. 

Some workers with low skill levels (also identified as being at increased risk) belong to this socio-professional category, and the result could be explained by financial constrains [22,23,25]. 

The time interval between diagnosis and first treatment might be another explanation. However, a recent meta-analysis found no difference in the time interval between diagnosis and first treatment across socio-economic positions [56]. Furthermore, some authors have suggested that treatment differences observed between social groups can influence cancer survival, with higher social groups receiving more efficient treatments and having a better survival rate [64]. In Switzerland, universal health insurance covers most of the expenses related to cancer treatment regardless of social status [65], however the indirect costs may be a burden for those with limited resources [23].

### 4.3. Methodological Considerations

The tumour stage at diagnosis was missing for 29% of women and 39% of men in our study sample despite an intensive effort to complete and confirm this data using cancer registry records. To properly manage missing values for this important variable, we used multiple imputations. The comparison of models using listwise deletion (complete case analyses) and those with imputed models yielded similar results. There is no evidence for biased estimates or insufficient precision due to imputation. 

Regarding the occupation variable, under the Akaike information criterion, the model with the occupation variable coded using one-digit ISCO-88 fitted the data better than the model with occupation coded using two-digit ISCO-88 codes (results not shown). Therefore, we used the former in our models. However, occupational groups at this large level of aggregation (1 or 2 digits) might not be specific enough to incorporate factors directly related to occupational exposures and working conditions. Previous studies showed that information on working conditions before and after diagnosis as well as information on return to work after cancer treatment are important to collect and analyse with respect to lung cancer survival. In Taiwan, lung cancer survivors who returned to work had significantly higher survival rates than those not returning to work, irrespective of the tumour stage at diagnosis [66]. This study also reported that patients who returned to work had a significantly reduced risk of all-cause mortality with a hazard ratio of 0.46 (95%-CI: 0.44–0.48), after controlling for age, treatment, income range, industrial classification, company size, and cancer stage. Other results confirmed that a better survival rate was seen in patients with stage III and IV lung cancer who returned to work [14]. In line with these findings, a systematic review revealed that lung cancer survivors in employment had a better quality of life regarding their physical functioning [13]. Conversely, the loss of a job has a negative impact on an individual’s well-being. Return to work facilitates physical recovery, maintains mental well-being, reduces economic hardship, and may contribute to patients’ recovery. Therefore, we believe that analyses at a finer level of ISCO-88 (3 or 4 digits), combined with information on the duration of sick leave, return to work, and working conditions could improve the understanding of net lung cancer survival. Having this information as well as information on smoking habits could also help explain the differences we observed between men and women, as well as the better survival of female workers with the second highest skill level compared to those with the highest level. Lung cancer incidence, mortality and survival in women are still rarely investigated and deserve more attention [67].

### 4.4. Strengths and Limitations

A major strength of our study lies in the use of the international classification of occupation that we also used to create the skill level required for the occupation. This allows for a common and replicable definition and measurement of occupation-related variables. In addition, our analyses were stratified by sex, which addresses the recommendation to improve occupational safety and health research by systematically including this factor in the analyses [51]. Moreover, the non-parametric method allowed us to calculate the net lung cancer survival for each of the three occupational variables without making any specific assumptions, whereas the parametric method allowed us to quantify the differences between groups in terms of hazard ratios and test the proportional hazards assumption. During the study period, completeness of case ascertainment has been high in western Swiss cancer registries, and the incidence information was of very good quality [68]. The use of a relative survival framework in our study was also appropriate to investigate inequalities in lung cancer survival. This allows accounting for disparities in mortality between study groups with respect to multiple causes of death [69]. Because of the association between occupational variables and mortality, we believe that future studies on occupational factors should also focus on relative survival methods.

In terms of limitations, occupation was missing for 67% of women and 48% of men in the SNC. An internal comparison showed that patients with and without occupational information had similar socio-demographic characteristics (results not shown). However, a comparison of the distribution of work-related variables for patients with and without information on occupation was not possible. Therefore, we could not fully rule out a selection bias. Assigning occupations as a time-dependent variable based on two time points could result in some misclassification, especially given that cancer survivors have a less stable employment trajectory than other workers [18]. Nevertheless, the information on the occupation at the time of the federal censuses is correct and we believe we assigned it accurately enough, since the majority of patients kept the same occupation between the two censuses [3]. Having occupational information at the time of diagnosis would be more accurate, but it was not available in the SNC. In a prior study, we assessed the quality of occupational data in all western Swiss cancer registries and in the SNC and concluded that quality was heterogeneous between registries [70]. To avoid differential misclassification of occupations, we chose to use SNC data rather than registry data. Moreover, we were constrained to aggregating the occupation under one-digit codes, reducing the variability of occupational situations, due to the limited number of observations per occupational group. This can explain a likely lack of statistical power in Models 2 and 3 and non-statically significant hazard ratios. Finally, information on smoking, duration of sick leave, and working conditions after return to work was not available to study their effect on cancer survival. Information on cancer treatment was also not available, but since the implementation of the Swiss federal law on cancer registration in 2020, all Swiss cancer registries should collect it systematically [71]. The use of this information in future large, nationwide, or international studies will allow a more accurate estimation of factors affecting net lung cancer survival.

Such studies are essential to informing health and social protection systems, which should guarantee appropriate work conditions for cancer survivors and educate them on their rights and obligations during sick leave [72]. It is important that clinicians and institutions consider work-related issues in cancer patients and perform adequate organizational and normative interventions, particularly in the most at-risk occupational groups.

## 5. Conclusions

This study reports the net survival for lung cancer across three occupation-related variables: occupation, skill level required for the occupation, and the socio-professional category of employment. We found that the net survival varied across skill levels and that the lowest skill level was associated with the worst survival prospects in both men and women. In the parametric models with a minimal adjustment, we identified several occupational groups at higher risk of mortality compared to the reference category, particularly among men. The adjustment for the histological type of lung cancer and tumour stage at diagnosis allowed us to control for the effect of these variables and indirectly control for smoking. After this adjustment, most hazard ratios remained higher than 1, though non-statistically significant. Workers in paid employment without specific information on occupation were identified as the most at-risk socio-professional category in nearly all models. As this study was conducted using a relatively small sample and limited set of covariates, further studies are required, taking into account smoking habits and treatments administrated to the patients. Information on return to work and working conditions before and after lung cancer diagnosis will also be highly valuable to analysing their effect on net lung cancer survival in large nationwide or international studies. Such studies are essential to informing health and social protection systems, which should guarantee appropriate work conditions for cancer survivors, beneficial for their quality of life and survival.

## Figures and Tables

**Table 1:** Description of the study sample: Incident lung cancer cases in French-speaking Switzerland (1990–2014).

Characteristics	Male		Female	
	*n*	(%)	*n*	(%)
Total	4360	(100)	1413	(100)
Nationality				
Swiss	3298	(78)	1226	(84)
Non-Swiss	1062	(22)	187	(16)
Civil status				
Single	409	(29)	219	(31)
Married	3330	(64)	735	(57)
Widowed	90	(1)	108	(3)
Divorced	531	(6)	351	(9)
Occupation				
Legislators, senior officials, and managers	480	(11)	101	(7)
Professionals	392	(9)	112	(8)
Technicians and associate professionals	721	(17)	287	(20)
Clerks	340	(8)	374	(26)
Service workers and shop and market sales workers	276	(6)	318	(23)
Skilled agriculture and fishery workers	178	(4)	15	(1)
Craft and related trades workers	1043	(24)	59	(4)
Plant and machine operators and assemblers	399	(9)	13	(1)
Elementary occupations	531	(12)	134	(9)
Skill level required for the occupation				
Low	531	(12)	134	(9)
Intermediate low	2236	(51)	779	(55)
Intermediate high	721	(17)	287	(20)
High	872	(20)	213	(15)
Socio-professional category				
Top management and independent professions	229	(5)	23	(2)
Other self-employed	827	(19)	174	(12)
Professionals and senior management	371	(9)	91	(6)
Supervisors/low level management and skilled labour	1927	(44)	744	(53)
Unskilled employees and workers	868	(20)	345	(24)
In paid employment, not classified elsewhere	138	(3)	36	(3)
Calendar period				
1990–1994	758	(17)	146	(10)
1995–1999	1268	(29)	297	(21)
2000–2004	691	(16)	222	(16)
2005–2009	752	(17)	312	(22)
2010–2014	891	(20)	436	(31)
Age at entry (years): mean ± standard deviation	60.7 ± 8.0		58.4 ± 8.5	
Duration of follow-up (years): mean ± standard deviation	2.5 ± 4.0		2.8 ± 4.1	

**Table 2:** Tumour characteristics: Incident lung cancer cases in French-speaking Switzerland (1990–2014).

Characteristics	Male		Female	
	*n*	(%)	*n*	(%)
Total	4360	(100)	1413	(100)
Tumour stage				
Stage I	464	(11)	184	(13)
Stage II	222	(5)	79	(6)
Stage III	575	(13)	191	(14)
Stage IV	1420	(33)	546	(39)
Missing information	1679	(39)	413	(29)
Histological subtype				
Adenocarcinoma	1426	(33)	685	(48)
Squamous cell carcinoma	1253	(29)	154	(11)
Large cell carcinoma	131	(3)	33	(2)
Small cell carcinoma	690	(16)	220	(16)
Other specified carcinoma	162	(4)	71	(5)
Unspecified malignant neoplasms	682	(16)	245	(17)
Unclassifiable	16	(0)	5	(0)

**Table 3:** Hazard ratios and associated 95%-confidence intervals for lung cancer relative survival by work-related variables among males aged 18–85 in French-speaking Switzerland (1990–2014).

Characteristics	Model 1 *		Model 2 **		Model 3 ***	
Occupation						
Legislators, senior officials, and managers	Ref.		Ref.		Ref.	
Professionals	1.09	0.93–1.27	1.04	0.89–1.21	0.97	0.82–1.14
Technicians and associate professionals	**1.15**	**1.00–1.31**	1.09	0.95–1.25	1.03	0.89–1.19
Clerks	1.11	0.94–1.30	1.07	0.91–1.26	1.02	0.86–1.20
Service workers and shop and market sales workers	**1.17**	**1.99–1.39**	1.14	0.96–1.35	1.13	0.94–1.35
Skilled agricultural and fishery workers	**1.23**	**1.01–1.50**	1.19	0.98–1.45	1.06	0.86–1.29
Craft and related trades workers	**1.14**	**1.01–1.30**	1.09	0.96–1.24	1.00	0.88–1.15
Plant and machine operators and assemblers	1.07	0.92–1.25	1.06	0.90–1.23	1.02	0.87–1.20
Elementary occupations	**1.21**	**1.05–1.40**	**1.19**	**1.03–1.38**	1.09	0.94–1.27
Skill level required for the occupation						
Low	Ref.		Ref.		Ref.	
Intermediate low	0.94	0.84–1.04	0.92	0.82–1.02	0.94	0.84–1.06
Intermediate high	0.95	0.83–1.07	0.91	0.80–1.04	0.95	0.82–1.08
High	**0.86**	**0.76–0.97**	**0.85**	**0.75–0.97**	0.90	0.79–1.03
Socio-professional category						
Top management and independent professions	Ref.		Ref.		Ref.	
Other self-employed	1.18	0.99–1.40	1.18	0.99–1.40	1.12	0.94–1.34
Professionals and senior management	0.91	0.75–1.11	0.89	0.73–1.08	0.91	0.74–1.11
Supervisors/low-level management and skilled labour	1.08	0.92–1.27	1.06	0.90–1.25	1.07	0.90–1.27
Unskilled employees and workers	1.08	0.91–1.29	1.07	0.90–1.28	1.05	0.88–1.26
In paid employment, not classified elsewhere	**1.43**	**1.13–1.82**	**1.37**	**1.08–1.74**	**1.36**	**1.05–1.76**

* Model 1 is adjusted for age, calendar period and registry; ** Model 2 is adjusted for age, calendar period, registry, and histological type; *** Model 3 is adjusted for age, calendar period, registry, histological type, and tumour stage at diagnosis. Statistically significant results are shown in bold.

**Table 4:** Hazard ratios and associated 95%-confidence interval for lung cancer relative survival by work-related variables among females aged 18–85 in French-speaking Switzerland (1990–2014).

Characteristics	Model 1 *		Model 2 **		Model 3 ***	
Occupation						
Legislators, senior officials, and managers	Ref.		Ref.		Ref.	
Professionals	0.89	0.65–1.22	0.96	0.70–1.32	0.85	0.62–1.18
Technicians and associate professionals	**0.72**	**0.55–0.94**	0.78	0.60–1.03	0.77	0.58–1.02
Clerks	0.86	0.66–1.10	0.91	0.71–1.18	0.88	0.67–1.16
Service workers and shop and market sales workers	0.85	0.65–1.10	0.89	0.69–1.16	0.86	0.65–1.13
Skilled agricultural and fishery workers	1.20	0.65–2.22	1.05	0.57–1.95	0.93	0.50–1.73
Craft and related trades workers	0.86	0.59–1.25	0.90	0.62–1.30	0.82	0.55–1.23
Plant and machine operators and assemblers	0.98	0.49–1.97	1.03	0.51–2.08	0.81	0.39–1.66
Elementary occupations	0.97	0.72–1.30	1.01	0.75–1.37	0.96	0.70–1.32
Skill level required for the occupation						
Low	Ref.		Ref.		Ref.	
Intermediate low	0.89	0.72–1.10	0.89	0.72–1.11	0.90	0.72–1.13
Intermediate high	**0.75**	**0.58–0.96**	**0.77**	**0.60–0.99**	0.80	0.61–1.04
High	0.97	0.75–1.25	0.97	0.75–1.25	0.96	0.73–1.25
Socio-professional category						
Top management and independent professions	Ref.		Ref.		Ref.	
Other self-employed	0.94	0.56–1.57	0.88	0.52–1.48	0.99	0.58–1.68
Professionals and senior management	0.81	0.47–1.41	0.74	0.43–1.29	0.84	0.48–1.49
Supervisors/low-level management and skilled labour	0.84	0.51–1.39	0.80	0.48–1.31	0.96	0.58–1.59
Unskilled employees and workers	0.88	0.53–1.45	0.87	0.52–1.44	1.04	0.62–1.74
In paid employment, not classified elsewhere	1.09	0.59–2.03	1.06	0.57–1.96	1.41	0.75–2.67

* Model 1 is adjusted for age, calendar period and registry; ** Model 2 is adjusted for age, calendar period, registry, and histological type; *** Model 3 is adjusted for age, calendar period, registry, histological type, and tumour stage at diagnosis. Statistically significant results are shown in bold.

## Data Availability

Due to the nature of this research, study participants could not agree to their data being shared publicly. Supporting data is therefore not available in accordance with ethical and legal requirements.
